# Effects of etomidate combined with dexmedetomidine on adrenocortical function in elderly patients: a double-blind randomized controlled trial

**DOI:** 10.1038/s41598-022-16679-1

**Published:** 2022-07-19

**Authors:** Fangjun Wang, Zheng Yang, Sisi Zeng, Luyue Gao, Jiabei Li, Na Wang

**Affiliations:** 1 The Affiliated Hospital of North Sichuan Medical College, Nanchong, China; 2 The North Sichuan Medical College, Nanchong, China

**Keywords:** Drug discovery, Health care, Medical research

## Abstract

Etomidate has been advocated to be used in anesthesia for the elderly and the critically ill patients due to its faint effect on cardiovascular system. But the dose-dependent suppression of etomidate on adrenal cortex function leads to the limitation of its clinical application. Clinical research showed that dexmedetomidine could reduce the dose requirements for intravenous or inhalation anesthetics and opioids, and the hemodynamics was more stable during the operation. The objective was to observe the effect of etomidate combined with dexmedetomidine on adrenocortical function in elderly patients. 180 elderly patients scheduled for elective ureteroscopic holmium laser lithotripsy were randomly allocated to PR group anesthetized with propofol-remifentanil, ER group anesthetized with etomidate-remifentanil, and ERD group anesthetized with dexmedetomidine combined with etomidate-remifentanil. Patients in each group whose operation time was less than or equal to 1 h were incorporated into short time surgery group (PR_1_ group, ER_1_ group and ERD_1_ group), and whose surgical procedure time was more than 1 h were incorporated into long time surgery group (PR_2_ group, ER_2_ group and ERD_2_ group). The primary outcome was the serum cortisol and ACTH concentration. The secondary outcomes were the values of SBP, DBP, HR and SpO_2_, the time of surgical procedure, the dosage of etomidate and remifentanil administered during surgery, the time to spontaneous respiration, recovery and extubation, and the duration of stay in the PACU. The Serum cortisol concentration was higher at t_1~2_   in ERD_1_ group compared to ER_1_ group (*P* < 0.05). The Serum cortisol concentration at t_1~3_   was higher in ERD_2_ group than in ER_2_ group (*P* < 0.05). The Serum ACTH concentration was lower at t_1~2_  in ERD_1_ group compared to ER_1_ group (*P* < 0.05). The Serum ACTH concentration at t_1~3_  was lower in ERD_2_ group compared to ER_2_ group (*P* < 0.05). The SBP at T_1_ and T_3_ were higher in ER_2_ and ERD_2_ group than in PR_2_ group (*P* < 0.05). The DBP in ER_1_ and ERD_1_ group were higher at T_1_ compared to PR_1_ group (*P* < 0.05). The dosage of etomidate was significantly lower in ERD_1_ group and ERD_2_ group than in ER_1_ group and ER_2_ group (*P* < 0.05), respectively. The administration of dexmedetomidine combined with etomidate can attenuate the inhibition of etomidate on adrenocortical function in elderly patients and maintain intraoperative hemodynamic stability.

## Introduction

Etomidate is a short acting intravenous general anaesthetic derived from imidazole, which shows better sedative effect and no analgesic effect. The drug has been advocated to be used in anesthesia for the elderly and the critically ill patients due to its faint effect on cardiovascular system. The adrenocortical function of the patients anaesthetized with etomidate was suppressed, and it was dose-dependent^1,2^. Dexmedetomidine is a highly effective and selective α 2-adrenergic receptor agonist, which manifests the effects of sedation, analgesia, anti-anxiety and sympathetic inhibition in a dose-dependent manner, with few side effects. At present, clinical research shows that dexmedetomidine can reduce the dose requirements for intravenous or inhalation anesthetics and opioids, and the hemodynamics is more stable during the operation^3–5^. However, there is no report on the effect of etomidate combined with dexmedetomidine on adrenocortical function in elderly patients. We hypothesized that dexmedetomidine could reduce the intraoperative dose requirements for etomidate, and the inhibition of adrenocortical function is also attenuated with the decrease in the dose of etomidate. Therefore, the aim of this study was to observe the effect of etomidate combined with dexmedetomidine on adrenocortical function in elderly patients.

## Methods

This study received approval from the Ethics Committee of the affiliated hospital of north sichuan medical college, Sichuan, China (Ref. 2018ER(R)008) in March 2018 and was registered at the Chinese Clinical Trial Registry (http://www.chictr.org.cn/; Registration number: ChiCTR1800015421, 29/03/2018). All participants provided written informed consent before participation. Patients scheduled for elective ureteroscopic holmium laser lithotripsy were enrolled. The inclusion criteria were age ≥ 60 years old, American Society of Anaesthesiologists physical status 1 or 2, a diagnosis of kidney or ureteral calculi. The exclusion criteria were as follows: severe functional liver or kidney disease, Cognitive dysfunction (performance < 26 points on a Montreal Cognitive Assessment), abnormal state of consciousness (including sleepiness, mental confusion, lethargic sleep and comatose), with a medical history of steroid therapy, with an endocrine disease. Withdrawal criteria: patients refusing to participate, change of surgical plan, incomplete data collection.

All patients enrolled were randomly divided into three groups, using sealed envelopes indicating the allocation, to receive intravenous anesthesia with propofol-remifentanil (PR group, *n* = 60), etomidate-remifentanil (ER group, *n* = 60) and etomidate-remifentanil combined with dexmedetomidine (ERD group, *n* = 60). Randomization was done by using the random number table, 180 three-digit numbers selected randomly from the random number table were serialized from small to large, then the serial numbers 1–60 were set as PR group, 61–120 as ER group and 121–180 as ERD group. All cards identifying patient grouping information were sealed in opaque envelopes. Randomization was performed by an anesthesiologist who was not responsible for surgical anesthesia of the patients or data collection. The anaesthesia nurses prepared the dexmedetomidine or saline according to the concealed envelope for random allocation. Patients in each group whose operation time was less than or equal to 1 h were incorporated into short time surgery group (PR_1_ group, ER_1_ group and ERD_1_ group), and whose operation time was more than 1 h were incorporated into long time surgery group (PR_2_ group, ER_2_ group and ERD_2_ group). The participating patients, surgeons, nurses and anaesthetists were blinded to the treatment allocation.

Patients were routine monitored with electrocardiography, noninvasive blood pressure (systolic blood pressure, mean arterial pressure and diastolic blood pressure), heart rate, respiratory rate, pulse oximetry, end-tidal CO2, the bispectral index and temperature upon arrival at the operating room. The 6 l/min oxygen was provided to all patients by a facemask. After a good upper extremity IV access secured, anaesthesia was induced with intravenous injection of midazolam 0.04 mg/Kg, propofol 1.5 mg/Kg, remifentanil 2 μg/Kg, cis-atracurium 0.2 mg/Kg in PR group, and midazolam 0.04 mg/Kg, etomidate 0.3 mg/Kg, remifentanil 2 μg/Kg, cis-atracurium 0.2 mg/Kg in ER group and ERD group. Controlled mechanical ventilation was adjusted to maintain an end-tidal carbon dioxide concentration of 35 to 45 mmHg after endotracheal tube insertion. Anaesthesia was maintained according to a BIS value of 40 to 60 with propofol plasma target concentration of 2 to 3 μg/ml and remifentanil plasma target concentration of 4 to 6 ng/ml in PR group, and etomidate plasma target concentration of 0.4 to 0.6 μg/ml and remifentanil plasma target concentration of 4 to 6 ng/ml in ER group and ERD group. Dexmedetomidine 0.4 μg/kg·h was administered immediately after induction of anesthesia in ERD group, and equal volume of normal saline was administered in the other groups. Cis-atracurium was administered according to intraoperative requirements in all groups. Atracurium and dexmedetomidine were stopped 45 min and 20 min before the end of the operation, respectively. Propofol, etomidate and remifentanil were stopped five minutes before the end of the operation. The patients were extubated after spontaneous respiration (tidal volume > 6 ml/kg, respiratory rate > 13/min), SpO_2_ > 90% under air inspiration, BIS > 80, and a train-of-four (TOF) ratio ≥ 0.9. Patients were transferred to the post-anesthesia care unit (PACU) after extubation, and when the modified Aldrete score > 9, the patients were transferred to the surgical ward. Hypotension (defined as systolic falling more than 20% before anesthesia or systolic values lower than 80 mmHg) was treated with ephedrine 6 mg intravenous bolus immediately. Bradycardia (defined as heart < 55 beats/minute) was treated with 0.5 mg of injection atropine.

6 ml venous blood of the patients was taken 15 min before anesthesia induction (t_0_), 6 h(t_1_), 12 h(t_2_), 24 h(t_3_), 48 h (t_4_) and 72 h (t_5_) after anesthesia respectively, all the blood samples were centrifugated at 3000 r/min for 5 min, 2 ml of serum of each sample was taken and stored at – 80 °C in refrigerator for detection later. The serum cortisol concentration was measured by electrochemiluminescence (ECL)^2^, and plasma adrenocorticotropic hormone (ACTH) was determined by radioimmunoassay^6^. The values of SBP, DBP, HR and SpO_2_ were recorded 5 min before anesthesia induction (T_0_), 5 min after anesthesia induction (T_1_), at the beginning of surgery (T_2_), during surgery (T_3_), 6 h after surgery (T_4_), 12 h after surgery (T_5_), 24 h after surgery (T_6_) and 48 h after surgery (T_7_). The time of surgical procedure, the dosage of etomidate and remifentanil administered during surgery, the time to spontaneous respiration, recovery and extubation (time from stopping administration of propofol or etomidate to spontaneous respiration, recovery and extubation respectively), and the duration of stay in the PACU were recorded.

### Statistical analysis

Statistical analyses were carried out using SPSS 19.0. Previous study found that 24 h after administration of etomidate, the plasma cortisol concentration of patients decreased about 4 µg/dl ^7^. In order to detect a difference of at least 2ug/dl in serum cortisol concentration between the two study groups with 90% power and 5% probability of type 1 error, this calculation assumed an SD of 2.2 in the serum cortisol concentration, 27 subjects were required per group. To account for a 10% dropout rate, 30 elderly patients in each group were recruited. The following formulas were used to compute the sample size:

$$n = \frac{{\left( {z_{\alpha } + z_{\beta } } \right)^{2} *2\sigma^{2} }}{{\delta^{2} }}$$

σ stands for standard deviation and δ represents the difference of the means.

Quantitative variables were expressed as mean ± standard deviation (SD), enumeration data was presented as frequencies. Comparison of the demographic data and clinical characteristics of the six groups were made using the Student’s t-test, Mann–Whitney *U* test and *x*^2^ test as appropriate. Repeated measures analysis of variance was used for comparisons of SBP, DBP, HR, serum cortisol and ACTH concentration levels among groups at each time point, if comparison between groups was positive, the SNK post hoc test was performed. The statistical significance was determined as *p* < 0.05.

### Statement of ethics

All procedures performed in studies involving human participants were in accordance with the ethical standards of the institutional and/or national research committee and with the 1964 Helsinki declaration and its later amendments or comparable ethical standards. Following the approval by the Ethics Committee of the affiliated hospital of north Sichuan Medical College (Ref. 2018ER(R)008), we obtained the written informed consent from all the participants for this randomized prospective clinical trial conducted at the affiliated hospital of north sichuan medical college, on patients with kidney or ureteral calculi.

## Results

**Figure 1:**
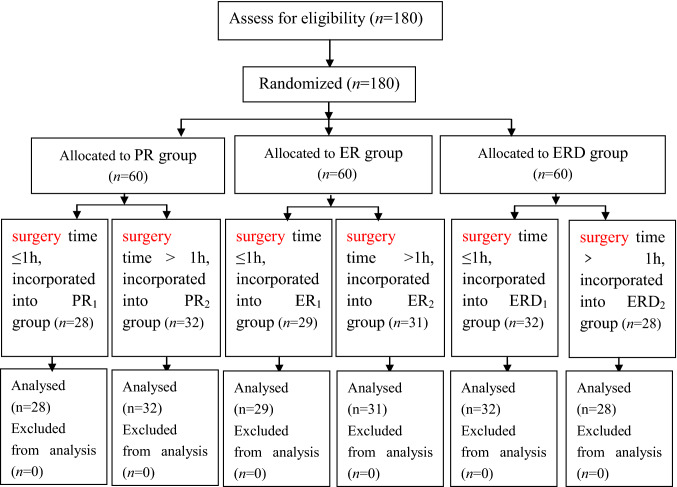
One hundred aneighty patients were screened for eligibility, and subsequently allocated to three groups. No patient dropped out of the trial. A total of one hundred and eighty patients completed the study (in this figure).

One hundred and eighty patients were screened for eligibility, and subsequently allocated to three groups. No patient dropped out of the trial. A total of one hundred and eighty patients completed the study (shown in Fig. 1).

There were no differences in age, weight, ASA grade and sex ratio among patients in each group (shown in Tables 1 and 2).

**Table 1:** Demographic data in short operation time groups.

Patient characteristics	PR_1_ *n* = 28	ER_1_ *n* = 29	ERD_1_ *n* = 32	*F/X*^2^ values
Sex (male/female)	15/13	14/15	16/16	0.0691
Age (years)	66.4 ± 4.6	65.6 ± 3.1	67.3 ± 4.8	1.21
Weight (kg)	57.5 ± 5.8	56.9 ± 7.1	58.0 ± 6.4	1.03
ASA (I/II)	9/19	11/18	13/19	0.4488

The Patient characteristics in short operation time groups are shown in this table. Patient characteristics were similar among the three groups, (in this table).

Values are mean ± SD. *ASA* American Society of Anesthesiologists, *PR*_*1*_ Propofol-remifentanil, *ER*_*1*_ Etomidate-remifentanil, *ERD*_*1*_ Etomidate-remifentanil and dexmedetomidine.

**Table 2:** Demographic data in long operation time groups.

Patient characteristics	PR_2_ *n* = 32	ER_2_ *n* = 31	ERD_2_ *n* = 28	*F/X*^2^ values
Sex (male/female)	15/17	15/16	15/13	0.2588
Age (years)	65.7 ± 4.0	65.2 ± 3.5	66.1 ± 3.8	0.51
Weight (kg)	56.9 ± 7.0	56.6 ± 8.8	58.7 ± 9.0	0.52
ASA (I/II)	14/18	12/19	11/17	0.1293

The Patient characteristics in long operation time groups are shown in this table. Patient characteristics were similar among the three groups, (in this table).

Values are mean ± SD. *ASA* American Society of Anesthesiologists, *PR*_*2*_ Propofol-remifentanil, *ER*_*2*_ Etomidate-remifentanil, *ERD*_*2*_ Etomidate-remifentanil and dexmedetomidine.

**Figure 2:**
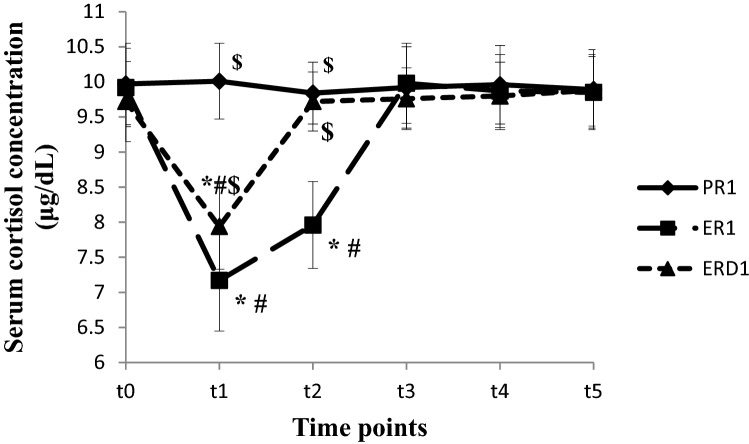
The Serum cortisol concentration changes in the short operation time groups at different time points. The Serum cortisol concentration at different time points in short operation time groups are shown in this figure. The Serum cortisol concentration were lower at t_1~2_ in ER1 group and t_1_ in ERD_1_ group compared to t_0_ and PR_1_ group (*P* < 0.05). The Serum cortisol concentration were higher at t_1~2_ in ERD_1_ group compared to ER_1_ group (*P* < 0.05), (in this figure).

**Figure 3:**
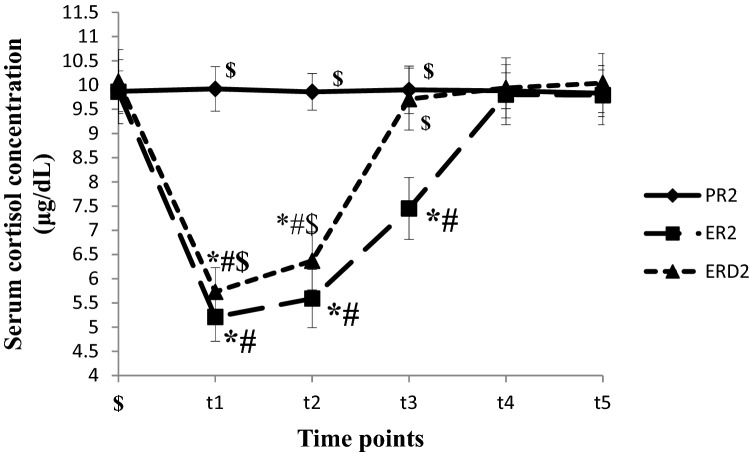
The Serum cortisol concentration changes in the long operation time groups at different time points. The Serum cortisol concentration at different time points in long operation time groups are shown in this figure. The Serum cortisol concentration were lower at t_1~3_ in ER_2_ group and t_1~2_ in ERD_2_ group compared to t_0_ and PR_2_ group (*P* < 0.05). The Serum cortisol concentration were higher at t_1~3_ in ERD_2_ group compared to ER_2_ group (*P* < 0.05), (in this figure).

The Serum cortisol concentration was lower at t_1~2_ in ER_1_ group and t_1_ in ERD_1_ group compared to t_0_ and PR_1_ group (*P* < 0.05). The Serum cortisol concentration at t_1~2_ was higher in ERD_1_ group than in ER_1_ group (*P* < 0.05), (shown in Fig. 2). The Serum cortisol concentration was lower at t_1~3_ in ER_2_ group and t_1~2_ in ERD_2_ group compared to t_0_ and PR_2_ group (*P* < 0.05). The Serum cortisol concentration at t_1~3_ was higher in ERD_2_ group than in ER_2_ group (*P* < 0.05), (shown in Fig. 3).

**Figure 4:**
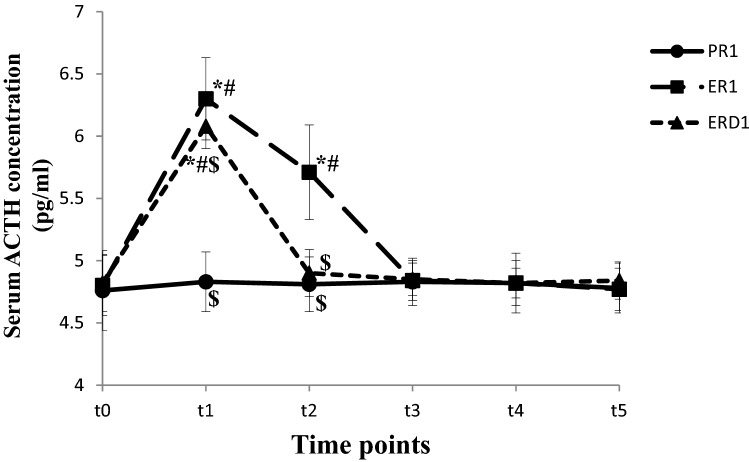
The Serum ACTH concentration changes in the short operation time groups at different time points. The Serum ACTH concentration at different time points in short operation time groups are shown in this figure. The Serum ACTH concentration were higher at t_1~2_ in ER_1_ group and t_1_ in ERD_1_ group compared to t_0_ and PR_1_ group (*P* < 0.05). The Serum ACTH concentration were lower at t_1~2_ in ERD_1_ group compared to ER_2_ group (*P* < 0.05), (in this figure).

**Figure 5:**
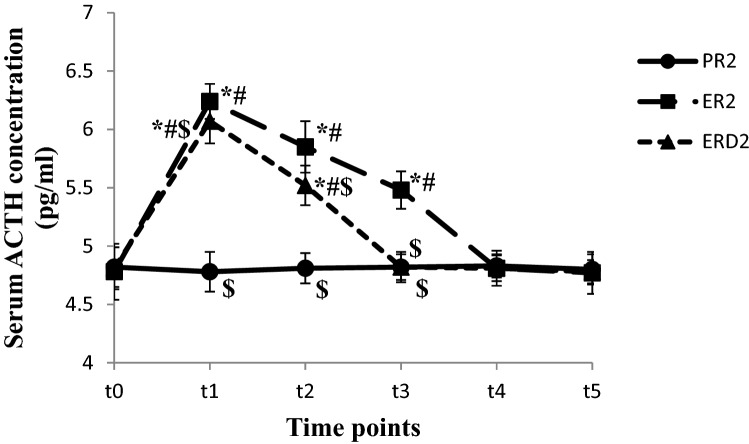
The Serum ACTH concentration changes in the long operation time groups at different time points. The Serum ACTH concentration at different time points in long operation time groups are shown in this figure. The Serum ACTH concentration were higher at t_1~3_ in ER_2_ group and t_1~2_ in ERD_2_ group compared to t_0_ and PR_2_ group (*P* < 0.05). The Serum ACTH concentration were lower at t_1~3_ in ERD_2_ group compared to ER_2_ group (*P* < 0.05) (in this figure).

The Serum ACTH concentration was higher at t_1~2_ in ER_1_ group and t_1_ in ERD_1_ group compared to t_0_ and PR_1_ group (*P* < 0.05). The Serum ACTH concentration at t_1~2_ was lower in ERD_1_ group than in ER_1_ group (*P* < 0.05), (shown in Fig. 4). The Serum ACTH concentration was higher at t_1~3_ in ER_2_ group and t_1~2_ in ERD_2_ group compared to t_0_ and PR_2_ group (*P* < 0.05). The Serum ACTH concentration at t_1~3_ was lower in ERD_2_ group than in ER_2_ group (*P* < 0.05), (shown in Fig. 5).

The SBP was lower at T_1_ compared to T_0_ in short time surgery groups (*P* < 0.05). The SBP in ER_1_ and ERD_1_ group was higher at T_1_and T_3_ compared to PR_1_ group (*P* < 0.05). The SBP at T_4_ was lower in ERD_1_ group than in ER_1_ group (*P* < 0.05), (shown in Table 3). The SBP was lower at T_1_ compared to T_0_ in long time surgery groups (*P* < 0.05). The SBP in ER_2_ and ERD_2_ group were higher at T_1_ and T_3_ compared to PR_2_ group (*P* < 0.05). The SBP at T_4_ were lower in ERD_2_ group than in ER_2_ group (*P* < 0.05), (shown in Table 4).

**Table 3:** The SBP (mmHg) at T_0_, T_1_, T_2_, T_3_, T_4_, T_5,_ T_6_ and T_7_ in short operation time groups.

Time points	PR_1_ *n* = 28	ER_1_ *n* = 29	ERD_1_ *n* = 32	*F* values	*P* values
T_0_	122.6 ± 11.8	123.4 ± 9.8	121.7 ± 11.7	0.22	0.8005
T_1_	93.8 ± 8.4*^$^	105.7 ± 6.7*^#^	103.0 ± 9.5*^#^	14.92	0.000
T_2_	115.3 ± 10.6*	116.5 ± 7.4*	115.0 ± 8.4*	0.55	0.5818
T_3_	112.5 ± 8.0*^$^	118.6 ± 6.3*^#^	117.6 ± 9.1*^#^	4.93	0.0095
T_4_	129.1 ± 8.6*	130.4 ± 10.0*	124.0 ± 7.0^#$^	5.2	0.0074
T_5_	120.3 ± 7.9	123.1 ± 6.9	121.2 ± 7.6	0.78	0.461
T_6_	119.8 ± 9.2	122.9 ± 10.4	121.7 ± 8.5	0.58	0.56
T_7_	121.7 ± 12.1	124.3 ± 10.3	122.6 ± 10.1	0.19	0.8285
*F* values	119.93	60.07	50.4	–	–
*P* values	0.000	0.000	0.000	–	–

The SBP at different time points in short operation time groups are shown in this table. The SBP were lower at T_1_compared to T_0_ in three groups (*P* < 0.05). The SBP in ER_1_ and ERD_1_ group were higher at T_1_ and T_3_ compared to PR_1_ group (*P* < 0.05). The SBP in ERD_1_ group were lower at T_4_ compared to ER_1_ groups (*P* < 0.05), (in this table).

Values are mean ± SD. *PR*_*1*_ Propofol-remifentanil, *ER*_*1*_ Etomidate-remifentanil, *ERD*_*1*_ Etomidate-remifentanil and dexmedetomidine, *T*_*0*_ before the induction of anesthesia, *T*_*1*_ 5 min after induction of anesthesia, *T*_*2*_ the beginning of operation, *T*_*3*_ during operation, *T*_*4*_ 6 h after surgery, *T*_*5*_ 12 h after surgery. *T*_*6*_ 24 h after surgery, *T*_*7*_ 48 h after surgery.*p < 0.05 vs. T_0_; ^#^*p* < 0.05 vs. PR_1_ group; ^$^*p* < 0.05 vs. ER_1_ group.

**Table 4:** The SBP (mmHg) at T_0_, T_1_, T_2_, T_3_, T_4_, T_5,_ T_6_ and T_7_ in long operation time groups.

Time points	PR_2_ *n* = 32	ER_2_ *n* = 31	ERD_2_ *n* = 28	*F* values	*P* values
T_0_	122.5 ± 9.2	121.3 ± 11.4	120.6 ± 10.9	0.14	0.8705
T_1_	95.3 ± 6.8*^$^	102.8 ± 8.0*^#^	104.5 ± 9.3*^#^	11.54	0.000
T_2_	115.1 ± 7.9*	118.3 ± 8.4	116.1 ± 9.8	1.09	0.3416
T_3_	113.5 ± 8.1*^$^	120.0 ± 8.5^#^	118.2 ± 10.1^#^	4.77	0.0108
T_4_	130.2 ± 9.8*	128.2 ± 10.4*	122.6 ± 9.5^#$^	4.31	0.0164
T_5_	120.3 ± 8.0	122.1 ± 9.1	119.8 ± 7.4	0.97	0.3817
T_6_	123.0 ± 9.2	122.5 ± 9.5	118.6 ± 8.0	2.14	0.1237
T_7_	124.0 ± 8.3	120.1 ± 10.3	123.2 ± 9.3	1.11	0.3357
*F* values	48	18.4	10.65	–	–
*P* values	0.000	0.000	0.000	–	–

The SBP at different time points in long operation time groups are shown in this table. The SBP were lower at T_1_compared to T_0_ in three groups (*P* < 0.05). The SBP in ER_2_ and ERD_2_ group were higher at T_1_ and T_3_ compared to PR_2_ group (*P* < 0.05). The SBP in ERD_2_ group were lower at T_4_ compared to ER_2_ groups (*P* < 0.05), (in this table).

Values are mean ± SD. *PR*_*1*_ Propofol-remifentanil, *ER*_*1*_ Etomidate-remifentanil, *ERD*_*1*_ Etomidate-remifentanil and dexmedetomidine, *T*_*0*_ before the induction of anesthesia, *T*_*1*_ 5 min after induction of anesthesia, *T*_*2*_ the beginning of operation, *T*_*3*_ during operation, *T*_*4*_ 6 h after surgery, *T*_*5*_ 12 h after surgery, *T*_*6*_ 24 h after surgery, *T*_*7*_ 48 h after surgery.*p < 0.05 vs. T_0_; ^#^*p* < 0.05 vs. PR_2_ group; ^$^*p* < 0.05 vs. ER_2_ group.

The DBP was lower at T_1_ compared to T_0_ in short time surgery groups (*P* < 0.05). The DBP in ER_1_ and ERD_1_ group was higher at T_1_ compared to PR_1_ group (*P* < 0.05). The DBP at T_4_ was lower in ERD_1_ group than in ER_1_ group (*P* < 0.05), (shown in Table 5). The DBP was lower at T_1_ compared to T_0_ in long time surgery groups (*P* < 0.05). The DBP in ER_1_ and ERD_1_ group was higher at T_1_compared to PR_1_ group (*P* < 0.05). The DBP at T_4_ was lower in ERD_1_ group than in ER_1_ group (*P* < 0.05), (shown in Table 6).

**Table 5:** The DBP (mmHg) at T_0_, T_1_, T_2_, T_3_, T_4_, T_5,_ T_6_ and T_7_ in short operation time groups.

Time points	PR_1_ *n* = 28	ER_1_ *n* = 29	ERD_1_ *n* = 32	*F* values	*P* values
T_0_	78.4 ± 5.8	77.6 ± 6.8	76.4 ± 6.8	0.76	0.4722
T_1_	56.1 ± 5.7*^$^	68.3 ± 9.8*^#^	67.1 ± 9.8*^#^	24.92	0.000
T_2_	76.9 ± 6.8	77.1 ± 7.9	77.9 ± 6.3	0.29	0.7491
T_3_	75.7 ± 6.7	78.2 ± 6.3	76.5 ± 7.1	1.19	0.3078
T_4_	85.1 ± 6.1*	84.9 ± 6.9*	80.9 ± 7.9*^#$^	4.22	0.0266
T_5_	77.8 ± 7.7	77.6 ± 6.4	78.3 ± 8.1	0.09	0.9135
T_6_	78.9 ± 4.0	78.6 ± 5.3	76.8 ± 4.2	1.68	0.1924
T_7_	77.6 ± 4.4	77.8 ± 5.7	77.3 ± 5.1	0.13	0.883
*F* values	55.52	10.8	9.88	–	–
*P* values	0.000	0.000	0.000	–	–

The DBP at different time points in short operation time groups are shown in this table. The DBP were lower at T_1_compared to T_0_ in three groups (*P* < 0.05). The DBP in ER_1_ and ERD_1_ group were higher at T_1_ compared to PR_1_ group (*P* < 0.05). The DBP in ERD_1_ group were lower at T_4_ compared to ER_1_ groups (*P* < 0.05), (in this table).

Values are mean ± SD. *PR*_*1*_ Propofol-remifentanil, *ER*_*1*_ Etomidate-remifentanil, *ERD*_*1*_ Etomidate-remifentanil and dexmedetomidine, *T*_*0*_ before the induction of anesthesia, *T*_*1*_ 5 min after induction of anesthesia, *T*_*2*_ the beginning of operation, *T*_*3*_ during operation, *T*_*4*_ 6 h after surgery, *T*_*5*_ 12 h after surgery. *T*_*6*_ 24 h after surgery. *T*_*7*_ 48 h after surgery.*p < 0.05 vs. T_0_; ^#^*p* < 0.05 vs. PR_1_ group; ^$^*p* < 0.05 vs. ER_1_ group.

**Table 6:** The DBP (mmHg) at T_0_, T_1_, T_2_, T_3_, T_4_, T_5,_ T_6_ and T_7_ in long operation time groups.

Time points	PR_2_ *n* = 32	ER_2_ *n* = 31	ERD_2_ *n* = 28	*F* values	*P* values
T_0_	77.2 ± 7.2	77.6 ± 9.1	76.8 ± 6.3	0.09	0.911
T_1_	56.5 ± 7.8*^$^	68.1 ± 6.2*^#^	67.3 ± 6.7*^#^	24.11	0.000
T_2_	77.1 ± 8.5	76.6 ± 8.7	77.2 ± 7.5	0.05	0.9471
T_3_	77.6 ± 6.9	77.9 ± 9.2	76.8 ± 7.7	0.25	0.783
T_4_	84.2 ± 6.8*	85.7 ± 5.7*	79.1 ± 7.2^#$^	2.59	0.081
T_5_	77.9 ± 7.6	77.2 ± 8.2	77.8 ± 7.6	0.09	0.9097
T_6_	78.7 ± 5.6	78.8 ± 5.9	78.4 ± 3.9	0.06	0.9405
T_7_	77.5 ± 5.8	78.4 ± 6.8	77.8 ± 5.3	0.2	0.8175
*F* values	41.37	12.57	8.69	–	–
*P* values	0.000	0.000	0.000	–	–

The DBP at different time points in long operation time groups are shown in this table. The DBP were lower at T_1_compared to T_0_ in three groups (*P* < 0.05). The DBP in ER_1_ and ERD_1_ group were higher at T_1_ compared to PR_1_ group (*P* < 0.05). The DBP in ERD_1_ group were lower at T_4_ compared to ER_1_ groups (*P* < 0.05), (in this table).

Values are mean ± SD. *PR*_*2*_ Propofol-remifentanil, *ER*_*2*_ Etomidate-remifentanil, *ERD*_*2*_ Etomidate-remifentanil and dexmedetomidine, *T*_*0*_ before the induction of anesthesia, *T*_*1*_ 5 min after induction of anesthesia, *T*_*2*_ the beginning of operation, *T*_*3*_ during operation, *T*_*4*_ 6 h after surgery, *T*_*5*_ 12 h after surgery, *T*_*6*_ 24 h after surgery, *T*_*7*_ 48 h after surgery.*p < 0.05 vs. T_0_; ^#^*p* < 0.05 vs. PR_2_ group; ^$^*p* < 0.05 vs. ER_2_ group.

The HR was lower at T_1_ and higher at T_4_ compared to T_0_ in short time surgery groups (*P* < 0.05). The HR in ERD_1_ group was lower at T_3_ and T_4_ compared to PR_1_ group and ER_1_ group (*P* < 0.05), shown in (shown in Table 7). The HR was lower at T_1_ compared to T_0_ in long time surgery groups (*P* < 0.05). The HR was higher at T_4_ compared to T_0_ in PR_2_ group and ER_2_ group, and lower at T_4_ compared to T_0_ in ERD_2_ group (*P* < 0.05). The HR in ERD_2_ group was lower at T_3_ and T_4_ compared to PR_2_ group and ER_2_ group (*P* < 0.05), (shown in Table 8).

**Table 7:** The HR (beats per minute) at T_0_, T_1_, T_2_, T_3_, T_4_, T_5,_ T_6_ and T_7_ in short operation time groups.

Time points	PR_1_ *n* = 28	ER_1_ *n* = 29	ERD_1_ *n* = 32	*F* values	*P* values
T_0_	79.6 ± 4.4	78.7 ± 6.5	77.3 ± 7.1	1.34	0.267
T_1_	69.3 ± 5.1*	67.7 ± 9.1*	66.5 ± 5.5*	1.08	0.344
T_2_	78.9 ± 6.4	77.0 ± 7.3	76.1 ± 7.1	0.81	0.4478
T_3_	77.3 ± 5.7	78.4 ± 7.6	67.5 ± 6.9* ^#$^	22.86	0.000
T_4_	84.5 ± 6.2*	85.3 ± 4.1*	81.4 ± 5.9* ^#$^	5.7	0.0048
T_5_	78.5 ± 8.0	78.8 ± 5.6	78.5 ± 6.7	0.25	0.777
T_6_	80.1 ± 7.1	78.6 ± 4.6	79.1 ± 5.2	1.3	0.2787
T_7_	79.1 ± 5.2	77.4 ± 5.1	78.4 ± 4.8	0.64	0.5291
*F* values	13.43	15.37	20.74	–	–
*P* values	0.000	0.0001	0.000	–	–

The HR at different time points in short operation time groups are shown in this table. The HR were lower at T_1_ and higher at T_4_ compared to T_0_ in three groups (*P* < 0.05). The HR in ERD_1_ group were lower at T_3_ and T_4_ compared to PR_1_ group and ER_1_ group (*P* < 0.05), (in this table).

Values are mean ± SD. *PR*_*2*_ Propofol-remifentanil, *ER*_*2*_ Etomidate-remifentanil, *ERD*_*2*_ Etomidate-remifentanil and dexmedetomidine, *T*_*0*_ before the induction of anesthesia, *T*_*1*_ 5 min after induction of anesthesia, *T*_*2*_ the beginning of operation, *T*_*3*_ during operation, *T*_*4*_ 6 h after surgery, *T*_*5*_ 12 h after surgery, *T*_*6*_ 24 h after surgery, *T*_*7*_ 48 h after surgery.*p < 0.05 vs. T_0_; ^#^*p* < 0.05 vs. PR_2_ group; ^$^*p* < 0.05 vs. ER_2_ group.

**Table 8:** The HR (beats per minute) at T_0_, T_1_, T_2_, T_3_, T_4_, T_5,_ T_6_ and T_7_ in long operation time groups.

Time points	PR_2_ *n* = 32	ER_2_ *n* = 31	ERD_2_ *n* = 28	*F* values	*P* values
T_0_	78.2 ± 5.6	79.1 ± 6.2	77.3 ± 4.5	0.81	0.4491
T_1_	65.7 ± 7.1*	66.3 ± 6.0*	67.8 ± 9.7*	0.64	0.5381
T_2_	79.4 ± 6.8	78.1 ± 7.8	76.7 ± 6.1	1.1	0.3374
T_3_	77.0 ± 6.3	77.1 ± 6.7	67.5 ± 5.2* ^#$^	22.34	0.000
T_4_	86.7 ± 4.2*	85.3 ± 5.2*	73.7 ± 7.2* ^#$^	46.46	0.000
T_5_	79.1 ± 5.8	79.6 ± 6.9	78.6 ± 5.5	0.11	0.8975
T_6_	78.3 ± 5.4	78.5 ± 4.9	77.8 ± 6.2	0.09	0.9168
T_7_	77.5 ± 4.9	77.5 ± 4.1	78.2 ± 9.1	0.13	0.8791
*F* values	55.38	23.5	11.59	–	–
*P* values	0.000	0.000	0.000	–	–

The HR at different time points in long operation time groups are shown in this table. The HR were lower at T_1_ compared to T_0_ in three groups (*P* < 0.05). The HR were higher at T_4_ compared to T_0_ in PR_2_ group and ER_2_ group, and lower at T_4_ compared to T_0_ in ERD_2_ group (*P* < 0.05). The HR in ERD_2_ group were lower at T_3_ and T_4_ compared to PR_2_ group and ER_2_ group (*P* < 0.05), (in this table).

Values are mean ± SD. *PR*_*2*_ Propofol-remifentanil, *ER*_*2*_ Etomidate-remifentanil, *ERD*_*2*_ Etomidate-remifentanil and dexmedetomidine, *T*_*0*_ before the induction of anesthesia, *T*_*1*_ 5 min after induction of anesthesia, *T*_*2*_ the beginning of operation, *T*_*3*_ during operation, *T*_*4*_ 6 h after surgery, *T*_*5*_ 12 h after surgery, *T*_*6*_ 24 h after surgery, *T*_*7*_ 48 h after surgery.*p < 0.05 vs. T_0_; ^#^*p* < 0.05 vs. PR_2_ group; ^$^*p* < 0.05 vs. ER_2_ group.

The duration of surgery and the length of stay in the PACU were similar among the three short time surgery groups. There was no difference in remifentanil dosage between the ER_1_ group and ERD_1_ group. The dosage of etomidate was significantly lower in ERD_1_ group compared with ER_1_ group (*P* < 0.05). The time to spontaneous respiration, tracheal extubation time and the time to recovery were longer in group ERD_1_ compared with group ER_1_ (*P* < 0.05), (shown in Table 9). The duration of surgery was similar among the three long time surgery groups. The dosages of remifentanil and etomidate were significantly lower in ERD_2_ group compared with ER_2_ group (*P* < 0.05). The time to spontaneous respiration, tracheal extubation time, the time to recovery and the PACU stay time were increased more significantly in group ERD_2_ compared with group ER_2_ (*P* < 0.05), (shown in Table 10).

**Table 9:** Clinical characteristics in short operation time groups.

Clinical characteristics	PR_1_ *n* = 28	ER_1_ *n* = 29	ERD_1_ *n* = 32	*F* values	*P* values
Duration of surgery (minute)	45.9 ± 7.1	46.6 ± 5.9	47.3 ± 6.1	0.11	0.8978
Dosage of etomidate (milligram)	–	54.2 ± 5.9	45.1 ± 5.6^$^	36.4	0.000
Dosage of remifentanil (microgram)	–	915.7 ± 41.2	897.9 ± 38.0	2.95	0.0911
Tme to spontaneous respiration (minute)	16.5 ± 1.7	17.2 ± 2.4	19.1 ± 2.5^#$^	10.84	0.000
Time to recovery (minute)	18.9 ± 2.1	19.7 ± 2.7	23.4 ± 2.6^#$^	30.24	0.000
Tracheal extubation time (minute)	20.6 ± 2.4	21.5 ± 2.9	26.4 ± 2.5^#$^	44.48	0.000
PACU stay time (minute)	59.9 ± 6.2	58.4 ± 5.4	62.1 ± 9.4	1.93	0.1511

The clinical characteristics in short operation time groups are shown in this table.The duration of surgery and the length of stay in the PACU were similar among the three groups. There was no difference in remifentanil dosage between the ER_1_ group and ERD_1_group.The dosage of etomidate was significantly lower in ERD_1_ group compared with ER_1_ group (*P* < 0.05). The time to spontaneous respiration, tracheal extubation time and the time to recovery were significantly delayed in group ERD_1_ compared with group ER_1_ (*P* < 0.05), (in this table).

Values are mean ± SD. *PR* Propofol-remifentanil, *ER* Etomidate-remifentanil, *ERD* Etomidate-remifentanil and dexmedetomidine, *PACU* postanesthesia care unit. ^#^*p* < 0.05 vs. PR_1_ group; ^$^*p* < 0.05 vs. ER_1_ group.

**Table 10:** Clinical characteristics in long operation time groups.

Clinical characteristics	PR_2_ *n* = 32	ER_2_ *n* = 31	ERD_2_ *n* = 28	*F* values	*P* values
Duration of surgery (minute)	105.5 ± 20.6	102.9 ± 16.2	104.5 ± 19.7	0.54	0.5831
Dosage of etomidate (milligram)	–	95.0 ± 10.1	74.4 ± 7.1^$^	81.23	0.000
Dosage of remifentanil (microgram)	–	1676.5 ± 188.6	1452.9 ± 132.0^$^	25.55	0.000
Time to spontaneous respiration (minute)	17.4 ± 2.1	18.1 ± 2.7	19.9 ± 2.6^#$^	7.91	0.0007
Time to recovery (minute)	19.8 ± 2.1	20.3 ± 3.0	25.6 ± 2.8^#$^	41.61	0.000
Tracheal extubation time (minute)	21.3 ± 2.1	22.4 ± 3.2	27.75 ± 3.4^#$^	38.68	0.000
PACU stay time (minute)	69.4 ± 7.1	71.7 ± 9.9	77.7 ± 11.4^#$^	6.01	0.0036

The clinical characteristics in long operation time groups are shown in this table. The duration of surgery were similar among the three groups. The dosage of remifentanil and etomidate were significantly lower in ERD_2_ group compared with ER_2_ group (*P* < 0.05). The time to spontaneous respiration, tracheal extubation time, the time to recovery and the PACU stay time were longer in group ERD_2_ compared with group ER_2_ (*P* < 0.05), (in this table).

Values are mean ± SD. *PR*_*2*_ Propofol-remifentanil, *ER*_*2*_ Etomidate-remifentanil, *ERD*_*2*_ Etomidate-remifentanil and dexmedetomidine, *PACU* postanesthesia care unit. ^#^*p* < 0.05 vs. PR_2_ group; ^$^*p* < 0.05 vs. ER_2_ group.

## Discussion

In this study, we found that the plasma concentration levels of cortisol and ACTH returned to preoperative levels 24 h and 48 h after surgery in short time surgery group and long time surgery group, respectively. However, after administration of dexmedetomidine 0.4 μg/kg.h, the serum cortisol and ACTH concentrations returned to the preoperative level 12 h after surgery in short time surgery group, and 24 h after surgery in long time surgery group. The blood pressure during both induction of anaesthesia and surgery was more stable when anesthetized with etomidate than propofol, indicating that the elderly patients performed good hemodynamic stability when anesthetized with etomidate. After intravenous administration of dexmedetomidine, the recovery time was increased significantly especially in such short surgeries.

Although etomidate provides rapid onset, rapid recovery and reliable cardiovascular stability^2^, the suppressive effects of etomidate on adrenocortical function limits its clinical application by anesthetists, especially the increased mortality in critically ill patients was potentially due to the adrenal suppressive effects of etomidate^8^. A specific and reversible blockade of the 11α-hydroxylation and 11β-hydroxylation of adrenal steroid synthesis caused by etomidate lead to the decrease of cortisol, corticosterone and aldosterone synthesis^9^. It was found that the serum corticosterone concentration decreased significantly and lasted for more than 3 h after 120 min of etomidate infusion in rats^9^. Clinical studies found that when intravenous infusion of etomidate was used for anesthesia induction, the levels of plasma cortisol were suppressed in the first 6 h after induction by intravenous infusion of etomidate, and returned to pre anesthesia levels 24 h later^10^. The serum cortisol concentration of patients anesthetized with etomidate for electroconvulsive therapy for several times was decreased significantly at 24 h after each anesthesia, and returned to the preoperative level 48 h after anesthesia^11^. These studies suggested that the suppression of etomidate on adrenal cortical function was dose-dependent。In this study, etomidate 0.3 mg/kg was used for anesthesia induction, and the anesthesia was maintained with intravenous target concentration of etomidate 4 to 6 μg/ml. In the short time surgery group, the serum cortisol level was significantly lower compared to preoperative level at 6 to 12 h after surgery, and there was no significant difference in serum cortisol level between the baseline and 24 h after surgery. The plasma cortisol concentration was decreased more significantly at 6 to 24 h after surgery, and returned to the preoperative level at 48 h after surgery in the long time surgery group. The results showed that the adrenocortical function of the elderly patients anesthetized with etomidate was suppressed, and the suppression was also prolonged with the increase of anesthesia time.

Dexmedetomidine is a highly specific α_2_-adrenoreceptor agonist with short half-life period (about 2 h). It has a dose-dependent sedative and analgesic effect, and has no adverse effect on respiration^12^. The application of dexmedetomidine (0.5 g/kg) in pediatric patients anesthetized with sevoflurane could decrease the heart rate of children, but there were no significant changes in SBP, DBP or PETCO_2_^4^. It was shown that dexmedetomidine as an adjunct for inhalation anesthetics could effectively maintain the stability of circulation and respiration during surgery. In recent clinical trials, the effect of dexmedetomidine on the requirement for propofol and remifentanil in total intravenous anesthesia was studied. It was found that the administration of dexmedetomidine significantly decreased both the requirements for propofol and remifentanil during anesthesia induction and the dosage of propofol administered during surgery^3,13^. In this study, when dexmedetomidine was added to intravenous anesthesia with etomidate, the intraoperative dosages of etomidate were reduced by 17% and 22% in the short time surgery group and long time surgery group respectively, and the dosage of remifentanil was reduced by 13% in long time surgery group, which was consistent with the above research results.

In this study, the administration of dexmedetomidine not only reduced the etomidate requirements for total intravenous anesthesia in elderly patients, but also attenuated and shortened the inhibitory effect of etomidate on adrenocortical function in elderly patients. This is mainly because the inhibition effects of etomidate on adrenal cortex function were dose-dependent^14^, the administration of dexmedetomidine significantly reduced the requirement for etomidate, and with the reduction of etomidate dose, the inhibitory effect of etomidate on adrenal cortex was correspondingly attenuated in this study. In vitro studies showed that dexmedetomidine combined with etomidate had a stronger inhibitory effect on human adrenocortical cells than etomidate alone^15^. However, some scholars studied the effect of dexmedetomidine and etomidate on adrenocortical function in children and found that 3 h after induction of anesthesia, the serum cortisol concentration of patients in the etomidate group was the lowest, while there was no difference between dexmedetomidine group and the control group, indicating that dexmedetomidine had little or no effect on adrenocortical function^16^. The researches above showed that the inhibitory effect of dexmedetomidine on adrenal function is controversial currently. The inhibitory effect of dexmedetomidine on adrenocortical function in elderly patients was not studied in our study, so the effect of dexmedetomidine on adrenocortical function in elderly patients is still unknown.

In clinic, the induction of anesthesia with propofol often leads to the decrease of arterial blood pressure^17^. Due to the degradation of organ function and the declination of physiological function, it is easier to induce hypotension in elderly patients anesthetized with propofol^18^. The blood pressure was decreased significantly compared to the baseline in patients by using propofol and etomidate for anesthesia induction, and the decrease was greater in the propofol group compared to etomidate group^19^. In this study, 5 min after induction of anesthesia with propofol, the systolic pressure, diastolic pressure and heart rate were decreased by 23.5%, 28.4% and 13%, respectively. This was mainly attributed to propofol reducing cardiac output and systemic vascular resistance, and inhibiting baroreceptor reflex^18^. However, in the etomidate group, systolic blood pressure was decreased by 14.6%, diastolic blood pressure was decreased by 12% and heart rate was decreased by 14% 5 min after induction of anesthesia. Although there was no difference in the decrease of heart rate, the decrease of systolic and diastolic blood pressure was more gently in etomidate group compared to propofol group. Meanwhile, the blood pressure of patients during surgery was significantly lower in propofol group compared to etomidate group. It was suggested that the hemodynamic stability in the elderly patients could be better maintained with etomidate anesthesia.

Dexmedetomidine could maintain intraoperative hemodynamic stability by inhibiting sympathetic nervous system and attenuating the stress response^20^. Davy A et al.^21^ reported about 42% patients who were administered with dexmedetomidine developed various degree hypotension and bradycardia. Dexmedetomidine could decrease the heart rate and blood pressure in a dose- dependent manner^22^. A clinical study found that low dose dexmedetomidine (0.5u.kg-1.h-1) can effectively reduce the requirement of propofol and maintain the intraoperative hemodynamics of patients undergoing laparoscopic cholecystectomy^23^. Josephine et al.^24^ pointed out in their review on hemodynamic response of high- and low-dose dexmedetomidine that compared with high-dose dexmedetomidine, low-dose dexmedetomidine had better hemodynamic stability and shorter recovery time. In our study, there was no difference in systolic and diastolic blood pressure between etomidate alone group and combined dexmedetomidine group at 5 min after anesthesia induction, the beginning of surgery and during surgery. But the addition of dexmedetomidine could decrease the intraoperative heart rate more significantly than etomidate alone, and no patient developed bradycardia. It showed that although the combination of dexmedetomidine (0.4 µ/kg/h) could decrease intraoperative heart rate, but had little effect on the intraoperative blood pressure in elderly patients undergoing general anesthesia, which was consistent with the results of recent studies^23,25^.

There was little correlation between intraoperative dexmedetomidine and the recovery time after propofol anesthesia in common outpatient procedures, and the potential dose relationship was that the administration of per μg/kg dexmedetomidine would increase recovery time for about 15 min^26^. In present study, when etomidate was combined with dexmedetomidine, the time to spontaneous respiration, time to recovery and tracheal extubation time were prolonged. However, intraoperative intravenous infusion of dexmedetomidine (0.4 μ/kg/h) did not affect postoperative anesthesia recovery in patients undergoing thoracic surgery^27^, or provided faster recovery in patients undergoing tympanoplasty surgery^28^. It was suggested that the administeration of dexmedetomidine in long-term surgery rather than short-term surgery could provide faster recovery.

There are limitations in this study. Firstly, we didn’t design a trial to identify the effect of dexmedetomidine alone on adrenocortical function in elderly patients. It is not clear whether the administration of dexmedetomidine suppress the adrenocortical function. Secondly, an enzymatic block of 11-b-hydroxylase was demonstrated in a patient who received a prolonged infusion of etomidate^29^. We didn’t observe the enzymatic block of 11-b-hydroxylase both in short and long time surgery groups in this study, and the effects of intravenous infusion of dexmedetomidine combined with etomidate on the enzymatic block of 11-b-hydroxylase at different times need to be further studied. Thirdly, the principal adrenocortical products are cortisol, aldosterone and dehydroepiandrosterone sulphate^30^. We only observed changes in plasma concentrations of cortisol and adrenocorticotropin in present study, but the effect of administration of etomidate combined with dexmedetomidine on adrenocortical secretion of aldosterone was not clear. Fourthly, in our study, the surgery time of ureteral holmium laser lithotripsy was within 2.5 h, and the effect of etomidate combined with dexmedetomidine infusion for more than 2.5 h on adrenocortical function was unclear. We will apply the combination of dexmedetomidine and etomidate in long-term surgery to observe the changes of adrenal cortex function of elderly patients in future studies. Finally, the sample size of our study is too small. If this study had been performed on a larger sample size, there would probably have been more significant results in terms of the dose- and time-dependent effects of dexmedetomidine on etomidate-induced inhibition of adrenal cortical function.

In conclusion, the inhibitory effect of etomidate on adrenocortical function in elderly patients was prolonged with the duration of intravenous anesthesia with etomidate. The administration of dexmedetomidine combined with etomidate can attenuate the inhibition of etomidate on adrenocortical function in elderly patients and maintain intraoperative hemodynamic stability.
