# Author Correction: Effects of etomidate combined with dexmedetomidine on adrenocortical function in elderly patients: a double-blind randomized controlled trial

**DOI:** 10.1038/s41598-023-29104-y

**Published:** 2023-02-02

**Authors:** Fangjun Wang, Zheng Yang, Sisi Zeng, Luyue Gao, Jiabei Li, Na Wang

**Affiliations:** 1 The Affiliated Hospital of North Sichuan Medical College, Nanchong, China; 2 The North Sichuan Medical College, Nanchong, China

Correction to: *Scientific Reports* https://doi.org/10.1038/s41598-022-16679-1, published online 19 July 2022

The original version of this Article contained an error in the Methods section.

“Anaesthesia was maintained according to a BIS value of 40 to 60 with propofol plasma target concentration of 2 to 3 μg/ml and remifentanil plasma target concentration of 4 to 6 ng/ml in PR group, and etomidate plasma target concentration of 4 to 6 μg/ml and remifentanil plasma target concentration of 4 to 6 ng/ml in ER group and ERD group.”

now reads:

“Anaesthesia was maintained according to a BIS value of 40 to 60 with propofol plasma target concentration of 2 to 3 μg/ml and remifentanil plasma target concentration of 4 to 6 ng/ml in PR group, and etomidate plasma target concentration of 0.4 to 0.6 μg/ml and remifentanil plasma target concentration of 4 to 6 ng/ml in ER group and ERD group.”

The original Article has been corrected.

